# *PD-L1* polymorphism can predict clinical outcomes of non-small cell lung cancer patients treated with first-line paclitaxel-cisplatin chemotherapy

**DOI:** 10.1038/srep25952

**Published:** 2016-05-16

**Authors:** Shin Yup Lee, Deuk Kju Jung, Jin Eun Choi, Cheng Cheng Jin, Mi Jeong Hong, Sook Kyung Do, Hyo-Gyoung Kang, Won Kee Lee, Yangki Seok, Eung Bae Lee, Ji Yun Jeong, Kyung Min Shin, Seung Soo Yoo, Jaehee Lee, Seung Ick Cha, Chang Ho Kim, Jae Yong Park

**Affiliations:** 1 Lung Cancer Center, Kyungpook National University Medical Center, Daegu 702-201, Republic of Korea; 2 Departments of Internal Medicine, School of Medicine, Kyungpook National University, Daegu 700-842, Republic of Korea; 3 Department of Biochemistry and Cell Biology, School of Medicine, Kyungpook National University, Daegu 700-842, Republic of Korea; 4 BK21 Plus KNU Biomedical Convergence Program, Department of Biomedical Science, Kyungpook National University, Daegu 700-842, Republic of Korea; 5 Cell and Matrix Research Institute, School of Medicine, Kyungpook National University, Daegu 700-842, Republic of Korea; 6 Biostatistics Center, School of Medicine, Kyungpook National University, Daegu 700-842, Republic of Korea; 7 Department of Thoracic Surgery, School of Medicine, Kyungpook National University, Daegu 700-842, Republic of Korea; 8 Department of Pathology, School of Medicine, Kyungpook National University, Daegu 700-842, Republic of Korea; 9 Department of Radiology, School of Medicine, Kyungpook National University, Daegu 700-842, Republic of Korea

## Abstract

This study was conducted to investigate whether polymorphisms of genes involved in immune checkpoints can predict the clinical outcomes of patients with advanced stage non-small cell lung cancer (NSCLC) after 1st line paclitaxel-cisplatin chemotherapy. A total of 379 NSCLC patients were enrolled. Twelve single nucleotide polymorphisms (SNPs) of *PD-1, PD-L1*, and *CTLA-4* genes were selected and genotyped. The associations of SNPs with chemotherapy response and overall survival (OS) were analyzed. Among the 12 SNPs investigated, *PD-L1* rs2297136T > C and rs4143815C > G were significantly associated with clinical outcomes after chemotherapy. The rs2297136T > C was significantly associated with both better chemotherapy response and better OS, and the rs4143815C > G had a significantly better response to chemotherapy. Consistent with the individual genotype analyses, rs2297136C-rs4143815G haplotype (ht4) carrying variant alleles at both loci was significantly associated with better chemotherapy response and OS compared with combined other haplotypes. Patients with at least one ht4 had significantly better chemotherapy response and OS compared to those without ht4. *PD-L1* rs2297136T > C and rs4143815C > G polymorphisms may be useful for the prediction of clinical outcome of 1^st^ line paclitaxel-cisplatin chemotherapy in NSCLC. Further studies are needed to confirm our findings and to understand the role of *PD-L1* in the chemotherapy outcome of NSCLC patients.

Lung cancer is the leading cause of cancer deaths worldwide, with an average 5-year survival rate of 16%^1^. Non-small cell lung cancer (NSCLC), about two thirds of which present with advanced disease, comprises about 85% of primary lung cancers^2^. During the last decade, target agents such as EGFR-tyrosine kinase inhibitors or ALK inhibitors have led a major advance in the treatment of NSCLC^3^,^4^. However, the benefit has been confined to a subset of NSCLC patients who have an active target for the drugs. Platinum-based chemotherapy still plays an important role in the treatment of the majority of patients with advanced NSCLC, but provides only modest benefits with an objective response rate of 30–40% and median survival time of 8–11 months^5^,^6^. Treatment outcomes after chemotherapy vary widely among patients with similar clinical characteristics including stage, the best prognostic index for NSCLC. Therefore, there have been numerous investigations to identify molecular biomarkers that can better predict a patient’s clinical outcome.

Recently, immune checkpoints blockade has emerged as a novel therapy against cancer^7^. T cells are key players in host antitumor immune function: They can recognize cancer cells, generating cytotoxic T cell populations that can infiltrate the tumor and kill tumor cells. The checkpoints of T cell activation are crucial for maintaining self-tolerance and avoiding immune response-related tissue damage^7^. Cancer cells can take advantage of this inhibitory mechanism to evade host immune defense by expressing ligands which interact with co-inhibitory receptors on cytotoxic T-cells. Therefore, blocking the specific ligand or receptor may reactivate host immune responses and antitumor functions.

Cytotoxic T lymphocyte-associated antigen 4 (CTLA-4) is an inhibitory receptor which can downregulate T-cell activation at the priming phase. A monoclonal antibody blocking CTLA-4 from its ligands expressed on antigen presenting cells has been shown to improve overall survival (OS) in patients with advanced melanoma^8^. It has also been investigated in combination with paclitaxel and carboplatin for the treatment of NSCLC in a phase II clinical trial with a promising result^9^, prompting an ongoing phase III trial. Programmed cell death-1 (PD-1) pathway is a major immune checkpoint by which tumors may inactivate tumor-infiltrating lymphocytes (TILs) in the tumor microenvironment. PD-1 is expressed in various immune cell types including cytotoxic T-cells and its activation inhibits T-cell function, proliferation, and survival^10^,^11^. The PD-1 ligand, PD-L1 is expressed on activated T cells, B cells, dendritic cells, and macrophages, and can be induced in a variety of tissue types by inflammatory cytokines, such as IFN-γ^12^,^13^. PD-L1 expression has been reported in many types of cancer including lung cancer^14^,^15^,^16^. Antibody blockade against PD-1 and PD-L1 has shown substantial survival benefit in several types of cancer including NSCLC^17^,^18^,^19^,^20^,^21^. Notably, tumor PD-L1 expression by immunohistochemistry (IHC) has been found to be predictive of response to anti-PD-1 or anti-PD-L1 treatment^17^,^18^,^19^. In addition, the prognostic role of PD-L1 expression has also been investigated in different types of cancer, although the results are inconsistent^22^,^23^.

Because the host immune system plays an important role in killing cancer cells after chemotherapy, altered immune checkpoint function may influence chemotherapy outcomes. In addition, accumulating evidence indicates that cancer chemotherapy may modulate the immune function, which may enhance the effect of chemotherapeutic drugs^24^,^25^,^26^. For example, platinum drugs enhance T-cell activation by dendritic cells, and tumor cell recognition and killing by tumor-specific T cells through downregulation of PD-L1 and PD-L2^27^. In this study, we hypothesized that polymorphisms of genes involved in immune checkpoints may affect the antitumor immune activity, thereby influencing clinical outcomes of chemotherapy in patients with NSCLC. To test this hypothesis, we evaluated the association of polymorphisms in *PD-1, PD-L1*, and *CTLA-4* genes with the chemotherapy response and survival of NSCLC patients undergoing 1^st^ line paclitaxel-cisplatin chemotherapy.

## Results

### Patient Characteristics and Clinical Predictors

The association between clinicopathological characteristics and the chemotherapy response and OS are shown in Table 1. The overall response rate was 47.5%. We observed events (deaths) in 347 of the 379 patients (91.6%) and median survival time (MST) was 13.2 months (95% CI = 12.5–14.7 months). Only tumor histology was significantly associated with response to chemotherapy. The OS was significantly associated with age, gender, smoking status, tumor histology, weight loss, and second line chemotherapy (Table 1).

### The genotypes of rs2297136T > C and rs4143815C > G and clinical outcomes

**Figure 1:**
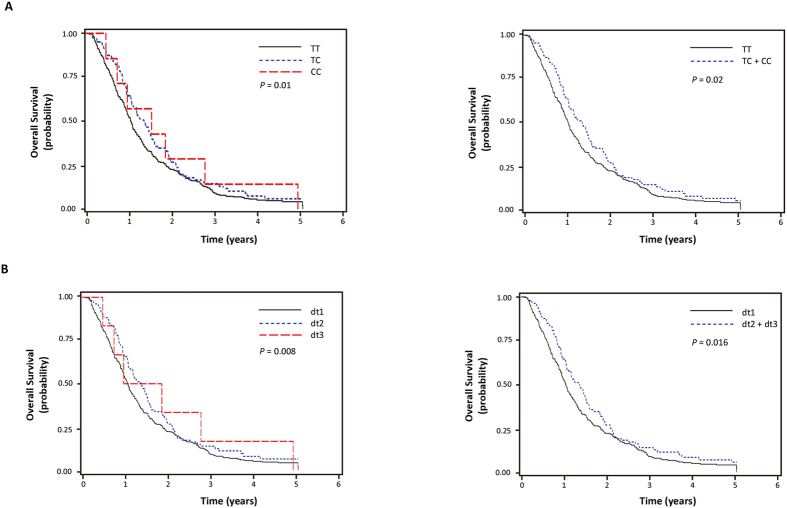
Kaplan-Meier plots of overall survival according to *PD-L1* rs2297136T > C (**A**), and diplotypes of rs2297136T > C and rs4143815C > G (**B**). *P* values in the multivariate Cox proportional hazard model.

The SNP ID, gene information, and minor allele frequencies are shown in Table 2. Of the 12 SNPs analyzed, the rs2297136T > C and rs4143815C > G SNPs in the 3′UTR of *PD-L1* were significantly associated with clinical outcomes after chemotherapy. The rs2297136T > C was significantly associated with both better chemotherapy response and better OS under additive model for the variant C allele (adjusted odds ratio [aOR] = 1.82, 95% CI = 1.19–2.77, *P* = 0.01; adjusted hazard ratio [aHR] = 0.76, 95% CI = 0.61–0.94, *P* = 0.01, respectively), and the rs4143815C > G was significantly associated with better response under additive model for the variant G allele (aOR = 1.42, 95% CI = 1.05–1.93, *P* = 0.02) although not significantly associated with OS (Table 3 and Fig. 1A).

### The haplotypes of rs2297136T > C and rs4143815C > G and clinical outcomes

The two SNPs, rs2297136T > C and rs4143815C > G, were in LD (|D’| = 0.9 and *r*^2^ = 0.22) with three predominant haplotypes accounting for 99.3% of the haplotypes in the subjects. Consistent with the individual genotype analyses, rs2297136C-rs4143815G haplotype (haplotype 4, ht4) carrying variant alleles at both loci was significantly associated with better chemotherapy response and OS compared with combined other haplotypes, rs2297136T-rs4143815C (ht1), rs2297136T-rs4143815G (ht2), and rs2297136C-rs4143815C (ht3) (aOR = 1.92, 95% CI = 1.27–2.91, *P* = 0.002; aHR = 0.76, 95% CI = 0.61–0.94, *P* = 0.01, respectively, Table 4). Therefore, we next examined the survival outcome of the patients carrying ht4. For this analysis, the diplotypes resulting from the four haplotypes were categorized into three groups according to the presence of zero, one, or two ht4 (i.e., diplotype 1 [dt1], ht1-3/ht1-3; dt2, ht1-3/ht4; dt3, ht4/ht4). The dt2 or dt3 with at least one ht4 had significantly better chemotherapy response and survival compared to the dt1 without ht4 (aOR = 2.13, 95% CI = 1.33–3.40, *P* = 0.002; aHR = 0.74, 95% CI = 0.58–0.95, *P* = 0.02, respectively, Table 4 and Fig. 1B).

### The predicted effect of rs2297136T > C and rs4143815C > G on the binding of miRNAs and *PD-L1* mRNA

**Figure 2:**
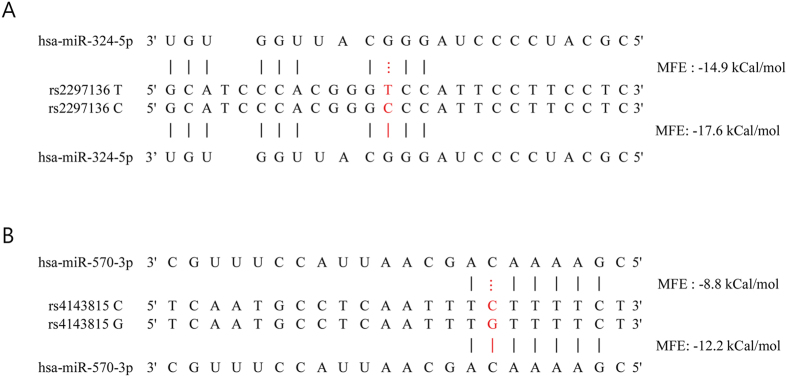
The predicted effect of allelic variants at *PD-L1* rs2297136T > C on hsa-miR-324-5p (**A**) and *PD-L1* rs4143815C > G on hsa- miR-570-3p (**B**) recognition. Minimum free energy (MFE) was predicted using RNAhybrid (http://bibiserv.techfak.uni-biele feld.de/rnahybrid/submission.html).

Using RNAhybrid2.2 (http://bibiserv.techfak.uni-bielefeld.de/rnahybrid/submission.html), we predicted minimum free energy (MFE) for the binding of miRNAs to *PD-L1* mRNA for both wild and variant alleles. The predicted effect of allelic variants at *PD-L1* rs2297136T > C and rs4143815C > G on miRNA binding was shown in Fig. 2. The MFE were −14.9 kCal/mol and −17.6 kCal/mol for the binding of miR-324-5p to rs2297136 T and C alleles, respectively, and −8.8 kCal/mol and −12.2 kCal/mol for the binding of miR-570-3p to rs4143815 C and G alleles, respectively. This result suggests that rs2297136T > C and rs4143815C > G may affect *PD-L1* expression by altered miRNA binding to 3′UTR of *PD-L1* mRNA.

## Discussion

Immune checkpoint blockade, targeting not the cancer cell itself but the host immune system, represents a paradigm shift in cancer therapy. It is evident that the interaction between tumors and their microenvironment including host immune system is critical not only in tumor development and progression, but also in the context of cancer therapy^24^. The intratumoral immune infiltrate indicative of an ongoing immune response has been correlated with therapeutic outcome^28^,^29^. In addition, systemic immune biomarkers such as polymorphisms in genes encoding immune modulators, e.g. interleukin-4 or -6, may reveal the way the host immune system responds to malignancies, thereby determining prognosis^30^,^31^. Likewise, genetic variations in immune checkpoint genes may be used as a potential predictor of cancer treatment outcome. This is the first study to investigate the effect of genetic polymorphisms in immune checkpoints on the outcomes of chemotherapy in treatment-naïve patients with advanced stage NSCLC. Two potentially functional polymorphisms, rs2297136T > C and rs4143815C > G, in *PD-L1* were significantly associated with 1^st^ line paclitaxel-cisplatin chemotherapy response and/or survival in NSCLC. The SNPs of *PD-L1* may be used to predict the clinical outcomes of NSCLC patients receiving 1^st^ line chemotherapy, thereby helping to identify subgroups of patients who would benefit from chemotherapy and to save patients from unnecessary toxicities.

In the present study, *PD-L1* rs2297136T > C and rs4143815C > G genotypes and their haplotypes were associated with clinical outcomes of paclitaxel-cisplatin chemotherapy in NSCLC. Because *PD-L1* rs2297136T > C and rs4143815C > G are located in 3′-UTR, the SNPs may cause a change in miRNA binding and PD-L1 expression. Functional prediction of the SNPs using RNAhybrid suggested that rs2297136T > C and rs4143815C > G may affect PD-L1 expression by modulating the miRNA-mRNA interaction. In addition, the rs4143815C > G has been reported to alter binding efficiency of miR-570 to *PD-L1* mRNA^32^. Altered PD-L1 expression may affect immune checkpoint function, which consequently may influence chemotherapy outcomes because host immune system plays an important role in killing cancer cells after chemotherapy. Cancer chemotherapy mainly targets tumor cells, but accumulating evidence indicates that it also affects the immune system^25^,^26^. Although immunosuppressive properties of chemotherapy are well known, many anticancer agents stimulate tumor-specific immune responses either by inducing immunogenic cancer cell death or by modulating immune effector mechanisms, which may boost host immunity and the effect of chemotherapy^24^,^25^. Apoptosis, a non-immunogenic cell death modality, is a frequent mechanism of chemotherapy-induced tumor cell death. However, many chemotherapeutic drugs can elicit specific cellular responses that cause tumor cell death to be immunogenic, leading to T cell mediated tumor-specific immune responses^25^,^33^. In addition, some anticancer drugs reportedly have immunomodulatory functions that contribute to the anticancer effect of the drugs. For example, paclitaxel has been reported to specifically impair cytokine production and viability in CD4^+^ FOXP3^+^ regulatory T cells but not in CD4^+^ FOXP3^−^ effector T cells, independent of Toll-like receptor 4 signaling^34^. In addition, platinum-based chemotherapeutics not only stimulate class I HLA expression but also inhibit signal transducer and activator of transcription 6 (STAT6)-regulated expression of PD-L2, thus limiting immunosuppression by both dendritic cells and tumor cells^35^. Given the immunological consequence of chemotherapy as a mechanism of killing cancer cells, the host immune system may play important roles in determining chemotherapy outcomes. Recent studies reported that the presence of TILs predicts response to neoadjuvant chemotherapy^36^. In addition, the presence of TILs has been correlated to PD-L1 expression in the tumor microenvironment as well as in tumor cells^37^, suggesting an association between PD-L1 expression in response to immune infiltrate and the outcome of chemotherapy. Taken together, immune checkpoint may be a potential determinant of the clinical outcome of chemotherapy. Our findings suggest that the PD-1/PD-L1 pathway plays a critical role in determining the response to chemotherapy and survival in NSCLC, and that PD-L1 polymorphisms may be predictive of the chemotherapy outcome in NSCLC, which is biologically plausible.

In conclusion, polymorphisms in the *PD-L1* gene were found to be independent predictive biomarkers for clinical outcomes of NSCLC patients receiving 1^st^ line paclitaxel-cisplatin chemotherapy. The SNPs of *PD-L1* may be useful in helping to refine therapeutic decisions in the treatment of NSCLC. Further studies with a larger population are needed to confirm our findings and to understand the role of PD-L1 in determining the clinical outcome of anticancer therapy.

## Methods

### Study populations

The study population has been described in our previous study^38^. In brief, 379 patients with stage III or IV NSCLC, who received at least two cycles of paclitaxel-cisplatin chemotherapy as a first-line treatment at Kyungpook National University Hospital (KNUH) in Daegu, Korea between August 2005 and December 2008, were enrolled. Patients who underwent radiotherapy concurrently with chemotherapy as a first treatment modality were excluded to avoid the confounding effect of radiation on the response to chemotherapy. The chemotherapy regimen included paclitaxel 175 mg/m^2^ administered i.v. over 3 h, and cisplatin 60 mg/m^2^ infused over 60 min given on day 1, every 3 weeks. Treatment was discontinued in case of disease progression, major toxicities, or according to patient’s or physician’s decision. Assessment of tumor response was carried out by computed tomography scan every two cycles. Responses were assessed using Response Evaluation Criteria in Solid Tumors^39^. The best overall response for each patient was reported and all responses were reviewed by an independent radiologist. Patients with a complete response (CR) or a partial response (PR) were defined as responders, and patients having stable disease (SD) or progressive disease (PD) were defined as non-responders. For the assessment of survival outcomes, OS, defined as the time between the date of chemotherapy start and the date of death or last follow-up, were recorded. Genomic DNA samples from the patients were provided by the National Biobank of Korea, KNUH, which is supported by the Ministry of Health, Welfare and Family Affairs. Written informed consent was obtained from all patients. This study was approved by the institutional review board of the KNUH and carried out in accordance with the institutional review board-approved guidelines.

### Selection of SNPs and genotyping

To collect potentially functional polymorphisms in *PD-1, PD-L1*, and *CTLA-4* genes, we searched the public database (http://www.ncbi.nlm.nih.gov/SNP) and related literature. The SNP selection favored those located in the promoter or untranslated region or coding region of the gene, those previously evaluated in relation to cancer, or those with evidence of functional significance. A total of 12 SNPs with the minor allele frequency (MAF) ≥0.05 in the HapMap JPT data were selected after excluding those in linkage disequilibrium (LD, *r*^2^ ≥0.8). Genotyping was performed using SEQUENOM’s MassARRAY^®^ iPLEX assay (SEQUENOM Inc., San Diego, CA) for 11 SNPs other than rs4143815, which was genotyped using the TaqMan^®^ assay (Applied Biosystems, Foster City, CA).

### Statistical analysis

Hardy-Weinberg equilibrium was tested using a goodness-of-fit χ^2^ test with 1 *degree of freedom*. The linkage disequilibrium among polymorphisms was measured by using HaploView (http://broad.mit.edu/mpg/haploview). The haplotypes and their frequencies were estimated using the Phase program^40^. The genotypes for each SNP were analyzed as a three-group categorical variable, and also analyzed under dominant, recessive, and additive genetic models. The association between clinical variables or genotypes and chemotherapy response was tested by odds ratio (OR) and 95% confidence intervals (CIs) using unconditional logistic regression analysis. Kaplan-Meier method was used to calculate survival estimates. The difference in OS according to different clinical variables or genotypes was compared using log-rank tests. Cox’s proportional hazard regression model was used for the multivariate survival analyses. The hazard ratio (HR) and 95% confidence interval (CI) were also estimated. A cut-off *p*-value of 0.05 was adopted for all the statistical analyses. The statistical data were obtained using Statistical Analysis System for Windows, version 9.2 (SAS Institute, Cary, NC, USA).

## Additional Information

**How to cite this article**: Lee, S. Y. *et al. PD-L1* polymorphism can predict clinical outcomes of non-small cell lung cancer patients treated with first-line paclitaxel-cisplatin chemotherapy. *Sci. Rep.* **6**, 25952; doi: 10.1038/srep25952 (2016).

## Figures and Tables

**Table 1:** Univariate analysis for response to chemotherapy and overall survival by clinical variables.

Variables	No. of	Response to chemotherapy				Overall survival				
		responders	nonresponders	OR (95% CI)	*P*	MST	95% CI	Log-Rank	HR (95% CI)	*P*
	cases	(CR + PR)	(SD + PD)			(months)		*P*		
Overall	379	180 (47.5)^a^	199 (52.5)			13.2	12.5–14.7			
Age (years)										
<65	179	93 (52.0)	86 (48.0)	1.00		15.7	13.7–17.7		1.00	
≥65	200	87 (43.5)	113 (56.5)	0.71 (0.48–1.07)	0.10	11.9	10.8–13.2	0.003	1.38 (1.11–1.70)	0.003
Gender										
Male	309	153 (49.5)	156 (50.5)	1.00		12.8	11.9–14.3		1.00	
Female	70	27 (38.6)	43 (61.4)	0.64 (0.38–1.09)	0.10	16.8	12.8–22.6	0.02	0.73 (0.56–0.96)	0.02
Smoking status										
Never	63	27 (42.9)	36 (57.1)	1.00		19.6	13.7–30.4		1.00	
Ever	316	153 (48.4)	163 (51.6)	1.25 (0.73–2.16)	0.42	12.8	11.7–14.2	0.001	1.59 (1.20–2.12)	0.002
Histological type										
Squamous cell ca.	184	98 (53.3)	86 (46.7)	1.00		13.2	11.7–14.4		1.00	
Adenoca.	172	71 (41.3)	101 (58.7)	0.62 (0.41–0.94)	0.02	15.1	12.1–17.5		0.74 (0.59–0.92)	0.01
NSCLC-NOS	23	11 (47.8)	12 (52.2)	0.80 (0.34–1.92)	0.62	11.4	7.4–12.9	0.01	1.25 (0.80–1.99)	0.34
Clinical stage										
III	159	82 (51.6)	77 (48.4)	1.00		14.7	12.8–17.4		1.00	
IV	220	98 (44.6)	122 (55.5)	0.75 (0.50–1.14)	0.18	12.7	10.8–14.2	0.12	1.18 (0.96–1.47)	0.12
PS ECOG										
0–1	310	149 (48.1)	161 (51.9)	1.00		14.1	12.6–15.7		1.00	
2	69	31 (44.9)	38 (55.1)	0.88 (0.52–1.49)	0.64	12.6	9.7–13.2	0.42	1.12 (0.85–1.48)	0.42
Weight loss										
No	233	116 (49.8)	117 (50.2)	1.00		14.4	12.5–16.6		1.00	
Yes	146	64 (43.8)	82 (56.2)	0.79 (0.52–1.19)	0.26	12.9	11.6–14.0	0.001	1.47 (1.18–1.83)	0.001
2^nd^ line Chemotherapy										
No	132					11.0	8.1–12.8		1.00	
Yes	247					15.1	13.2–16.6	0.02	0.76 (0.61–0.95)	0.02
Radiation to tumor										
No	340					12.9	11.6–14.3		1.00	
Yes	39					18.5	14.0–23.9	0.19	0.80 (0.57–1.12)	0.19

Abbreviation: OR, odds ratio; MST, median survival time; CI, confidence interval; HR, hazard ratio; PS, performance status; ECOG, Eastern Cooperative Oncology Group.

^a^Row percentage.

**Table 2:** Summary of twelve SNPs and response to chemotherapy and overall survival.

ID No.^a^	Gene	location	Alleles	CR (%)	MAF	HWP	*P* for response^b^			*P* for overall survival^c^		
							dominant	recessive	additive	dominant	recessive	additive
rs2297136	*PD-L1*	3′UTR	TC	99.2	0.16	0.35	0.01	0.20	0.01	0.02	0.15	0.01
rs4143815	*PD-L1*	3′UTR	CG	96.6	0.40	0.67	0.21	0.01	0.02	0.47	0.18	0.23
rs822336	*PD-L1*	promoter	GC	99.5	0.26	0.73	0.67	0.11	0.31	0.68	0.95	0.72
rs822337	*PD-L1*	promoter	TA	96.6	0.34	0.88	0.75	0.16	0.36	0.30	0.27	0.19
rs822338	*PD-L1*	intron	CT	98.9	0.44	0.60	0.87	0.35	0.67	0.56	0.34	0.35
rs36084323	*PD1*	promoter	GA	97.6	0.47	0.31	0.13	0.19	0.08	0.99	0.42	0.62
rs2227982	*PD1*	missence	CT	98.9	0.46	0.15	0.20	0.09	0.07	0.96	0.53	0.74
rs10204525	*PD1*	3′UTR	AG	99.2	0.25	0.38	0.33	0.09	0.15	0.76	0.60	0.66
rs231775	*CTLA-4*	missence	GA	99.2	0.28	0.52	0.97	0.26	0.61	0.71	0.93	0.80
rs5742909	*CTLA-4*	promoter	CT	97.9	0.12	0.26	0.41	0.58	0.58	0.98	0.11	0.74
rs733618	*CTLA-4*	promoter	AG	98.7	0.45	0.91	0.37	0.80	0.47	0.46	0.70	0.48
rs11571316	*CTLA-4*	promoter	CT	98.2	0.16	0.56	0.91	0.34	0.86	0.89	0.95	0.91

Abbreviation: CR, call rate; MAF, minor allele frequency; and HWE, Hardy-Weinberg equilibrium.

^a^Information about SNPs and SNP ID were obtained from NCBI database (http://ncbi.nih.gov).

^b^*P* values were calculated by multivariate regression analysis, adjusted for age, gender, smoking status, tumor histology, stage, ECOG performance status, and weight loss.

^c^P-values were calculated using multivariate Cox proportional hazard models, adjusted for age, gender, smoking status, tumor histology, stage, ECOG performance status, weight loss, 2nd line chemotherapy and radiation to primary tumor.

**Table 3:** Genotypes of rs2297136 and rs4143815 polymorphisms and their associations with the response to chemotherapy and overall survival.

Polymorphism/Genotype	Location	No. of case (%)^a^	Response to chemotherapy				Overall survival			
			Responders (%)^b^	Non-responders (%)^b^	OR (95% CI)^c^	*P*^c^	MST (95% CI)^d^	*L-R P*	HR (95% CI)^d^	*P*^d^
rs2297136^e^	3′UTR									
TT		264 (70.2)	115 (43.6)	149 (56.4)	1.00		12.6 (11.4–13.9)	0.19	1.00	
TC		105 (27.9)	60 (57.1)	45 (42.9)	1.79 (1.12–2.87)	0.01	16.3 (12.8–18.5)		0.77 (0.60–0.98)	0.04
CC		7 (1.9)	5 (71.4)	2 (28.6)	3.60 (0.66–19.8)	0.14	18.4 (5.7–33.3)		0.52 (0.24–1.12)	0.10
Dominant					1.87 (1.18–2.96)	0.01	16.7 (12.8–18.4)	0.07	0.75 (0.59–0.95)	0.02
Recessive					3.05 (0.56–16.6)	0.20	13.2 (12.5–14.7)	0.61	0.57 (0.26–1.23)	0.15
Additive					1.82 (1.19–2.77)	0.01			0.76 (0.61–0.94)	0.01
rs4143815^e^	3′UTR									
CC		133 (36.3)	58 (43.6)	75 (56.4)	1.00		13.5 (11.6–16.0)	0.71	1.00	
CG		172 (47.0)	78 (45.4)	94 (54.7)	1.10 (0.69–1.77)	0.69	13.2 (11.3–14.8)		0.97 (0.76–1.24)	0.81
GG		61 (16.7)	38 (62.3)	23 (37.7)	2.26 (1.19–4.28)	0.01	14.6 (11.9–19.4)		0.80 (0.57–1.11)	0.18
Dominant					1.33 (0.85–2.08)	0.21	13.3 (12.1–15.2)	0.88	0.92 (0.73–1.16)	0.47
Recessive					2.14 (1.20–3.82)	0.01	13.2 (12.0–14.8)	0.41	0.81 (0.60–1.10)	0.18
Additive					1.42 (1.05–1.93)	0.02			0.91 (0.78–1.06)	0.23

Abbreviation: OR, odds ratio; CI, confidence interval; MST, median survival time (months); *L-R P*, Log-rank *P*; HR, hazard ratio.

^a^Column percentage.

^b^Row percentage.

^c^OR, 95% CI, and their corresponding *P* values were calculated by multivariate regression analysis, adjusted for age, gender, smoking status, tumor histology, stage, ECOG performance status, and weight loss.

^d^HRs, 95% CIs and their corresponding *P*-values were calculated using multivariate Cox proportional hazard models, adjusted for age, gender, smoking status, tumor histology, stage, ECOG performance status, weight loss, 2nd line chemotherapy and radiation to primary tumor.

^e^Genotype failure in 3 cases for rs2297136T > C and 13 cases for rs4143815C > G.

**Table 4:** Response to chemotherapy and overall survival according to *PD-L1* rs2297136T > C and rs4143815C > G haplotypes and diplotypes.

	Response to chemotherapy					Overall survival		
	No.(%)^a^	Response	Non-response	OR (95% CI)^b^	*P*^b^	Log-rank *p*	HR (95% CI)^c^	*P*^c^
Haplotype								
ht1. TC	446 (58.8)	199 (44.6)	247 (55.4)	1.00		0.32	1.00	
ht2. TG	193 (25.5)	91 (47.2)	102 (52.9)	1.13 (0.80–1.60)	0.49		1.03 (0.86–1.23)	0.74
ht3. CC	5 (0.7)	1 (20.0)	4 (80.0)	0.31 (0.03–2.84)	0.30		0.97 (0.40–2.38)	0.95
ht4. CG	114 (15.0)	69 (60.5)	45 (39.5)	1.98 (1.29–3.04)	0.002		0.76 (0.61–0.95)	0.02
*P*_trend_					0.003			0.02
ht1-3	644 (85.0)	291 (45.2)	353 (54.8)	1.00		0.08	1.00	
ht4	114 (15.0)	69 (60.5)	45 (39.5)	1.92 (1.27–2.91)	0.002		0.76 (0.61–0.94)	0.01
Diplotype								
dt1(ht1-3/ht1-3)	271 (71.5)	115 (42.4)	156 (57.6)	1.00		0.18	1.00	
dt2(ht1-3/ht4)	102 (26.9)	61 (59.8)	41 (40.2)	2.08 (1.29–3.35)	0.003		0.77 (0.60–0.98)	0.04
dt3(ht4/ht4)	6 (1.6)	4 (66.7)	2 (33.3)	3.21 (0.55–18.73)	0.20		0.47 (0.20–1.08)	0.07
*P*_trend_					0.002			0.01
dt1	271 (71.5)	115 (42.4)	156 (57.6)	1.00		0.07	1.00	
dt2 + dt3	108 (28.5)	65 (60.2)	43 (39.8)	2.13 (1.33–3.40)	0.002		0.74 (0.58–0.95)	0.02

Abbreviation: HR, hazard ratio; CI, confidence interval; ht, haplotype; dt, diplotype.

tumor histology, stage, ECOG, weight loss, 2nd line chemotherapy and radiation to primary tumor.

^a^Column percentage.

^b^aORs, 95% CIs and their corresponding *P*-values described the risk of being a responder and were calculated by multivariate regression analysis, adjusted for age, gender, smoking status, tumor histology, stage, ECOG and weight loss.

^c^aHRs, 95% CIs and their corresponding *P*-values were calculated using multivariate Cox proportional hazard models, adjusted for age, gender, smoking status.
